# Editorial: Hot topic: anti-inflammatory drug discovery

**DOI:** 10.3389/fchem.2023.1339736

**Published:** 2023-12-15

**Authors:** Joana Silva, Gi-Sang Bae, Ahmed Esmat Abdel Moneim, Mohd Saeed, James David Adams, Mohamed L. Ashour

**Affiliations:** ^1^ Center for Marine and Environmental Sciences (MARE–IPLeiria), Peniche, Portugal; ^2^ Wonkwang University, Iksan, Republic of Korea; ^3^ Helwan University, Helwan, Egypt; ^4^ University of Hail, Hail, Saudi Arabia; ^5^ Independent Researcher, Benicia, United States; ^6^ Faculty of Pharmacy, Ain Shams University, Cairo, Egypt

**Keywords:** plant medicines, flavonoids, triterpenes, opioids, Benzodiazepines

It has been a pleasure to put together this Research Topic on anti-inflammatory drugs. So far, the response has been much better than expected. This Research Topic seems to have had a real impact. Hopefully, the search for new anti-inflammatory drugs will look more closely at the leads suggested here. The treatment of pain and chronic pain with topical preparations is another area that needs more research.

*Anti-inflammatory sesquiterpene and triterpene acids from Mesona procumbens Hemsley* Huang et al. Six triterpene acids were identified and found to have anti-inflammatory activity, including mesoeudesmol. These compounds have been overlooked in the past because of concerns about oral bioavailability. Oral use may improve gut inflammation in diseases such as Crohn’s disease. However, recent research has shown that topical treatment of pain and chronic pain is the most effective. Medicines made from this plant might be effective when applied to the skin. The source of these compounds is the plant *M. procumbens* (also known as *Platastoma palustre*), which is used for food and medicine in Asia. The very popular food grass jelly is made from this plant. Triterpene acids will become more important in future healthcare.

*Potential effect of luteolin, epiafzelechin, and albigenin on rats under cadmium-induced inflammatory insult: In silico and in vivo approach* Shahzadi et al. Luteolin, epiafzelechin, and albigenin were found to have anti-inflammatory activity in this study. Luteolin and epiafzelechin are flavonoids, and albigenin is a triterpenoid proanthocyanidin. The source of luteolin is *Senegalia senegal*, gum Arabic, which is used as a food and medicine. The source of albigenin is *Albezia lebbeck*, which is native to India and Southeast Asia where it is used as a traditional medicine. Epiafzelechin comes from *Senegalia chundra*, which is used for timber and medicine. The report details the anti-inflammatory activity of the compounds in a cadmium-based model of inflammation. Additionally, computer modeling data of cyclo-oxygenase 2 binding of the compounds are included.

*The immunosuppressive effects and mechanisms of loureirin B on collagen-induced arthritis in rats* Zou et al. Loureirin B was found to be anti-inflammatory in a rheumatoid arthritis model. The compound is a flavonoid that comes from the traditional Chinese medicine *Dracaena cochinchinensis*. A synthetic procedure was devised for loureirin B and derivatives that were used in the study. Loureirin B was found to decrease the expression of voltage-gated potassium channels Kv 1.3 on effector memory T cells. This resulted in the depression of inflammatory cytokine expression by these cells. Joint swelling and inflammation were found to decrease in a rheumatoid arthritis model. Memory T cells are abundant in the skin and may be of interest in future research.

*Efficacy and safety analysis of midazolam combined with dezocine sedation and analgesia colonoscopy in patients with inflammatory bowel disease: a prospective single-center open study* Chen et al. Midazolam and dezocine provide adequate sedation during colonoscopy. This is useful in a clinical setting where anesthesiologists may not be available. Dezocine is an opioid drug that was discontinued in the United States in 2011 because it causes more respiratory depression than some other opioids. It is used in combination with the benzodiazepine midazolam to induce a twilight sleep type of anesthesia. Dezocine is widely used in China and is considered to be safer than fentanyl, which is the drug of choice in the United States. More than 100,000 patients die from opioids in the United States yearly. A safer approach must be found.

*A review of FDA approved drugs and their formulations for the treatment of breast cancer* Chaurasia et al. This is a thorough review of anti-inflammatory and other drugs used in breast cancer. Breast cancer was diagnosed in 2.3 million women in 2022 and accounted for 685,00 deaths. A useful table of 39 drugs used in breast cancer provides details of each of them. New drug formulations, some of which are in clinical trials, are discussed.

